# Effects of sequential microhydration on thymine tautomerization

**DOI:** 10.1007/s00894-026-06916-z

**Published:** 2026-08-29

**Authors:** Murillo H. Queiroz, Tiago Vinicius Alves, Roberto Rivelino

**Affiliations:** 1 Departamento de Físico-Química, Instituto de Química, Universidade Federal da Bahia Rua Barão de Jeremoabo, 147, Salvador, Bahia, 40170-115 Brazil; 2 Instituto de Física, Universidade Federal da Bahia, Salvador, Bahia, 40210-340 Brazil

**Keywords:** H-bonds, DNA bases, Tautomerization, QTAIM analysis

## Abstract

**Context:**

The tautomerization of thymine between its keto and enol forms is a key event for spontaneous mutagenesis. While the intrinsic enol-to-keto conversion is kinetically hindered in the gas phase, the presence of a discrete microhydration environment can drastically alter this landscape. Our M06-2X free energy calculations indicate that the cluster with two water molecules provides the lowest Gibbs free energy barrier among the investigated hydrated systems. However, benchmark electronic calculations show that the energetic differences between the mono-, bi-, and trihydrated clusters are small. HOMA index and QTAIM analyses confirm that the third water molecule induces an offset between aromaticity and intermolecular stabilization, providing a structural basis for the observed kinetic stabilization of the rare tautomer in a hydrated environment.

**Method:**

In this work, we employ Hybrid-Meta DFT calculations to investigate the impact of sequential microhydration (0 to 3 water molecules) on the proton-transfer dynamics. Stable geometries for all stationary points (minima and transition states) were obtained through full optimization using the hybrid-meta M06-2X functional along with the def2-TZVP basis set. The nature of the stationary points was confirmed by harmonic frequency analysis. Furthermore, the normal modes associated with the imaginary frequencies were inspected to confirm that they correspond to the proton-transfer coordinate. Kinetic rate constants were calculated based on Transition State Theory (TST) at 298.15 K. Topological properties of the electronic density were obtained through QTAIM and NCI analyses.

**Supplementary Information:**

The online version contains supplementary material available at https://doi.org/10.1007/s00894-026-06916-z.

## Introduction

The high fidelity of DNA replication is a cornerstone of genetic stability, yet it is periodically challenged by the existence of rare nucleobase tautomers [1, 2]. Among these, the enol form of thymine is of particular interest due to its potential for inducing spontaneous point mutations through mispairing with guanine, leading to A:T → G:C transitions [3]. While the keto-enol equilibrium strongly favors the isolated keto form, the local environment, specifically the presence of explicit water molecules, can significantly influence the relative stability and interconversion rates of these species [4–7].

Several theoretical studies have indicated that microhydration can substantially alter the tautomeric equilibria, hydrogen-bond (H-bond) arrangements, and proton-transfer energetics of nucleobases and related pyrimidine systems [7–12]. In particular, water-assisted proton-transfer pathways have been proposed as an important mechanism for facilitating tautomerization processes that would otherwise present prohibitive barriers in the gas phase [6, 9, 13, 14].

In our previous study, QTAIM analyses revealed that H-bonded chains in microhydrated thymine clusters exhibit significant cooperative stabilization effects [15]. We further showed that the interaction of thymine with up to three water molecules modulates its electronic structure and strengthens the stability of specific hydration patterns through non-covalent interactions. Although the electronic and spectroscopic properties of these clusters have been extensively characterized, the proton-transfer pathway and its frequency within these cooperative networks remain elusive. Moreover, despite the growing body of research on hydrated nucleobase clusters, systematic investigations of sequential hydration effects on proton-transfer kinetics and transition-state properties in thymine systems are still comparatively scarce.

Understanding the kinetic stability of the enol tautomer is crucial because its mutagenic potential is directly tied to its residence time in the polymerase active site [16]. It has been suggested that the aqueous environment can act as a catalytic bridge, facilitating proton transfer through a proton-wire mechanism, which lowers the energy barriers that are otherwise prohibitive in the gas phase [17].

In the present work, we build upon our previous findings by investigating the proton-transfer mechanism in microhydrated thymine clusters. Using Hybrid-Meta DFT calculations, we explore how the cooperative effects previously described [15] translate into kinetic barriers and proton-wire organization. Furthermore, the Harmonic Oscillator Model of Aromaticity (HOMA) [18, 19] and the Quantum Theory of Atoms in Molecules (QTAIM) [20–22] are employed to provide a structural rationale for the observed kinetic trends, thereby linking π-conjugation to the kinetic stability of the rare tautomer.

## Computational methods

The present calculations were carried out using the Gaussian 09 [23] software package. All geometries corresponding to stationary points, including complexes, transition states (TS), and products, were located and fully optimized without symmetry constraints. The hybrid meta-GGA M06-2X functional [24] was employed for all calculations due to its superior performance in describing reaction barriers, H-bonding, and non-covalent interactions of biological systems. To ensure a high-level description of the electronic density and the hydration shell, the def2-TZVP [25] triple-zeta basis set was employed. Finally, NCI analysis [26] was applied to identify different regions of the gradient of the electronic density.

The nature of the stationary points was confirmed through harmonic frequency analysis. TSs were identified by a single imaginary frequency corresponding to the concerted proton movement. Kinetic parameters were evaluated at 298.15 K and 1.0 atm. Theoretical rate constants (*k*_TST_) for the proton jump were calculated based on Transition State Theory (TST) [27, 28]. All reported activation free energies (∆*G*^‡^) were obtained from harmonic vibrational thermochemical analysis at 298 K and 1 atm.

Furthermore, the structural aromaticity of the pyrimidine ring during the interconversion was quantified using the HOMA index [18, 29] and QTAIM analysis [20–22]. This index was calculated from the optimized bond lengths, providing a geometric measure of how the microhydration environment modifies the aromatic character of the different species.

## Results and discussion

### Energetics and tautomerization profile

**Fig. 1:**
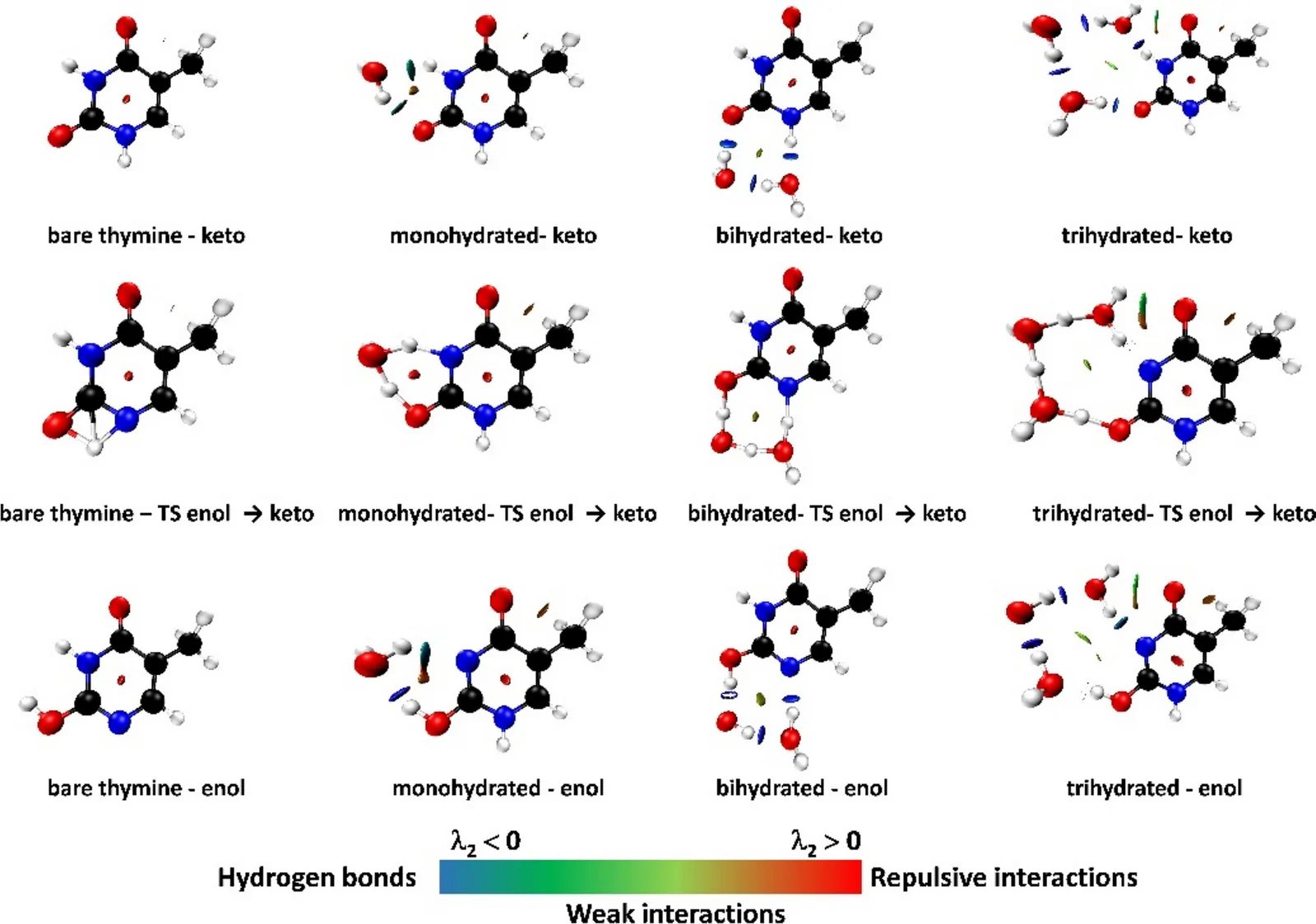
M06-2X/def2-TZVP optimized geometries and non-covalent interaction (NCI) isosurfaces (0.5 a.u.) for bare, bihydrated, and trihydrated complexes

The structural evolution of the thymine complexes upon sequential hydration is exhibited in Fig. 1, which displays the optimized geometries alongside the NCI isosurfaces. This topological analysis is useful to visualize the stabilization regions that are not immediately apparent from bond lengths alone.

As indicated in the NCI plots, the transition from thymine to the mono, bi, and trihydrated complexes establishes a highly efficient proton-wire characterized by strong, localized H-bonds (blue isosurfaces). In the trihydrated system, the additional water molecule introduces broader, delocalized stabilization regions (green isosurfaces), indicating a more complex and rigid non-covalent network. This structural reorganization is the physical basis for the kinetic modulation observed in our energetic calculations. At this point, it is important to mention that because of the complexity of the potential energy surface associated with microhydrated thymine clusters, the investigated hydrated arrangements were selected based on their energetic relevance and relative stability.

**Fig. 2:**
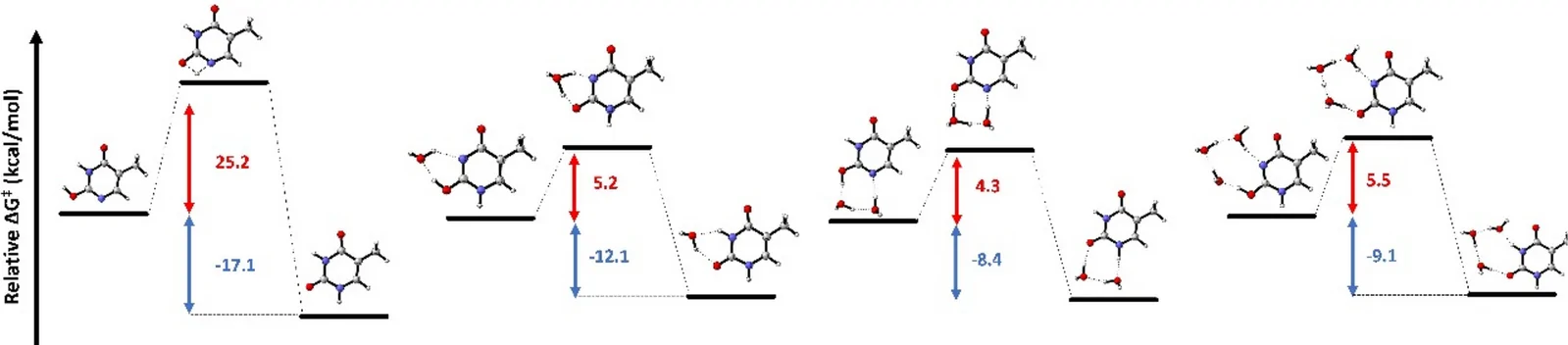
Gibbs free energy profile for the enol-to-keto tautomerization of thymine calculated at the M06-2X/def2-TZVP level. The reaction pathways are shown for bare thymine (left), the monohydrated complexes (center-left), the bihydrated complexes (center-right), and the trihydrated complexes (right)

The energy profile for the enol-to-keto conversion is significantly modified by the presence of a microhydration environment. As shown in Fig. 2, the activation barrier for the bare thymine is prohibitive at room temperature, i.e., 25.2 kcal/mol, resulting in a negligible *k*_TST_, i.e., ~ 10^–6^ s^−1^. The addition of a single water molecule already reduces the activation barrier to 5.2 kcal/mol, indicating the formation of an efficient water-assisted proton-transfer pathway. This reduction indicates that even a single water molecule is sufficient to alter the proton-transfer kinetics by enabling an H-bond-assisted pathway.

At the M06-2X level, the proton wire with two water molecules yields the lowest Gibbs free energy barrier (4.3 kcal/mol). However, benchmark electronic calculations of activation barriers indicate that the energetic differences among the hydrated systems are modest and slightly method dependent (see Table S1). However, the addition of a third water molecule increases the barrier to 5.5 kcal/mol, suggesting a stabilization of the enol complex that slightly hinders the reversion. Additional benchmark single-point calculations at the MP2/def2-TZVP and CCSD(T)/def2-TZVP levels, performed on the M06-2X optimized geometries, show that the energetic ordering of the hydrated clusters depends somewhat on the electronic structure method. Nevertheless, all methods predict similarly low electronic activation barriers for the hydrated systems. The complete benchmark data are provided in the Supporting Information. Table 1 reports the calculated activation free energies, imaginary frequencies, Wigner tunneling corrections, and rate constants for the water-assisted proton transfer in thymine.

**Table 1:** Calculated activation free energies ∆G^‡^ (in kcal/mol), activation enthalpies ∆H^‡^ (in kcal/mol), activation entropic term − *T*∆*S*^‡^ (in kcal/mol), imaginary frequencies |*υ*_imag_| (in cm^−1^), Wigner tunneling correction factors (*κ*_Wigner_), and corrected rate constants (*k*_corrected_) for the water-assisted tautomerization in thymine

System	∆*G*^‡^	∆*H*^‡^	− *T*∆*S*^‡^	|υ_imag_|	*κ*_Wigner_	*k*_TST_	*k*_corrected_
Bare	25.2	24.6	0.6	1892.4i	4.48	2.07 × 10^−6^	9.28 × 10^−6^
Monohydrated	5.2	3.6	1.6	1396.6i	2.89	8.99 × 10^8^	2.60 × 10^9^
Bihydrated	4.3	2.0	2.3	1150.7i	2.29	4.49 × 10^9^	1.03 × 10^10^
Trihydrated	5.5	2.6	2.9	648.1i	1.41	6.16 × 10^8^	8.67 × 10^8^

The considerable reduction in the activation barrier from the bare system to the bihydrated cluster (i.e., 25.2 to 4.3 kcal/mol) highlights the efficiency of the proton-wire mechanism. In the absence of water, the proton must undergo a strained intramolecular shift, characterized by a very high imaginary frequency (i.e., *υ* = 1892.4i cm^−1^). This high value is indicative of a very narrow and steep potential energy surface (PES) at the TS. The pronounced reduction in both the activation barrier and imaginary frequency upon sequential hydration suggests a transition from a localized intramolecular proton transfer to a water-assisted proton-transfer mechanism. Furthermore, within the M06-2X decomposition of the activation free energies into enthalpic (Δ*H*^‡^) and entropic (−*T*Δ*S*^‡^) contributions, the bihydrated complex exhibits the lowest Δ*G*‡ among the investigated systems at the M06-2X level.

Interestingly, although hydration reduces the enthalpic component of the activation barrier, the entropic contribution progressively increases with the number of water molecules. This trend suggests that larger hydrated clusters require greater structural arrangements to reach the TS geometry, resulting in increasingly unfavorable entropic costs. In this sense, the bihydrated complex appears to provide a favorable balance between enthalpic stabilization and entropic cost among the investigated systems.

**Fig. 3:**
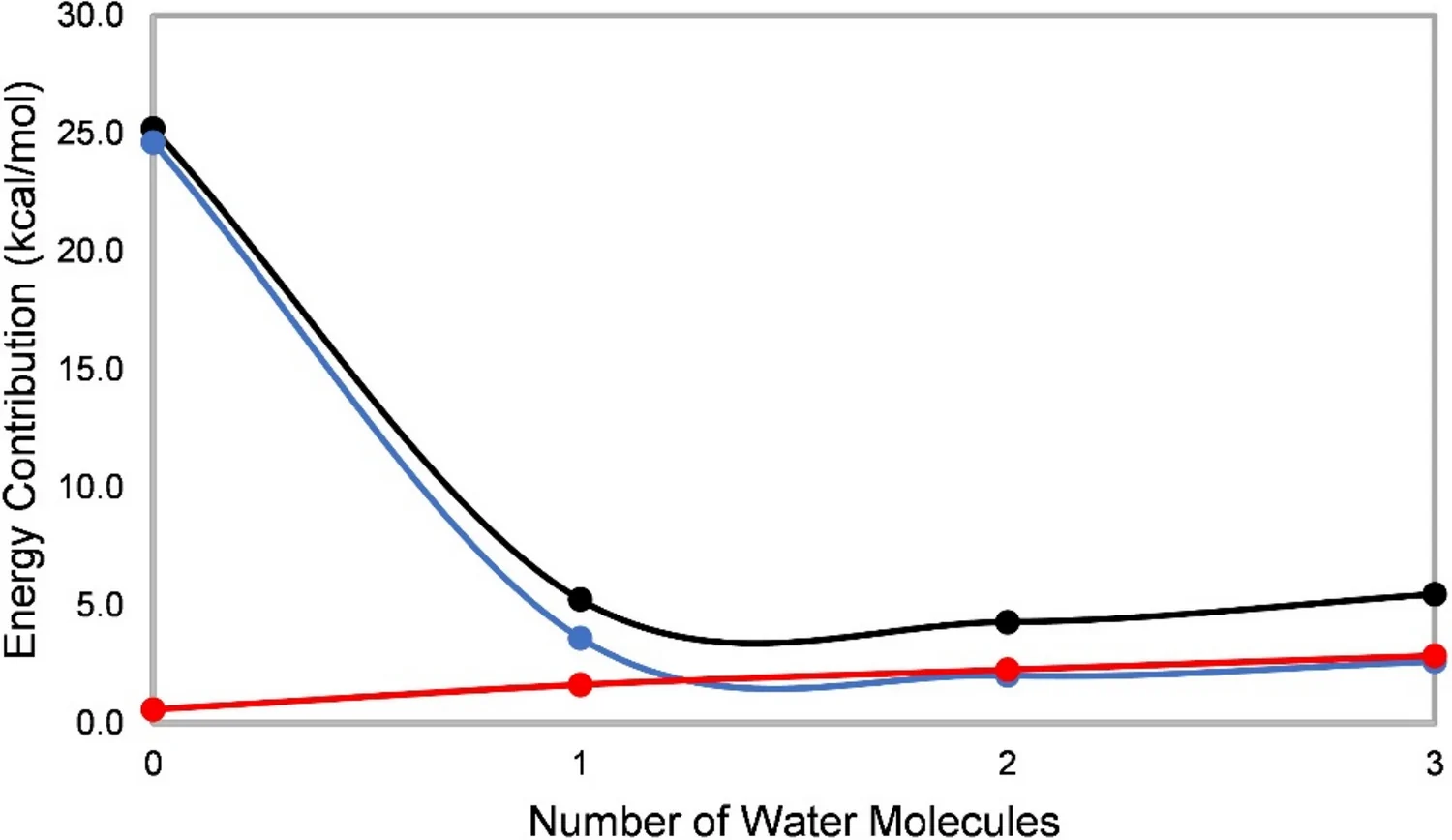
Variation of the activation free energy (Δ*G*^‡^), activation enthalpy (Δ*H*^‡^), and entropic contribution (− *T*Δ*S*^‡^) along the sequential hydration of thymine calculated at the M06-2X/def2-TZVP level. Curves in black, blue, and red are related to Δ*G*^‡^, Δ*H*^‡^ and − *T*Δ*S*^‡^, respectively

Furthermore, the evolution of the enthalpic and entropic contributions along the hydration sequence is summarized in Fig. 3. While hydration strongly lowers the enthalpic component of the activation barrier, the entropic contribution progressively increases with the number of water molecules. At the M06-2X level, the bihydrated system exhibits the lowest values for both Δ*H*^‡^ and Δ*G*^‡^, whereas the trihydrated complex presents a larger entropic contribution, which partially offsets the enthalpic stabilization. These results reinforce that the proton-transfer energetics are governed by a balance between enthalpic stabilization and the increasing structural reorganization required to reach the transition-state geometry in the more hydrated clusters.

Upon the introduction of water molecules, a significant shift in the reaction dynamics is observed. The imaginary frequency systematically decreases from 1892.4i cm^−1^ in the bare system to 1396.6i cm^−1^, 1150.7i cm^−1^, and finally 648.1i cm^−1^ for the mono-, bi-, and trihydrated complexes, respectively. This trend reveals that the hydration shell not only lowers the peak of the barrier but also structurally relaxes the TS, resulting in a broader and flatter PES. Furthermore, the Wigner tunneling correction factors also progressively decrease along the hydration sequence, indicating that quantum tunneling effects become less pronounced as the proton-transfer barrier becomes broader and less localized. This behavior is consistent with the progressive flattening of the proton-transfer PES, since narrower and steeper barriers generally favor larger tunneling contributions, whereas broader barriers tend to reduce tunneling effects. This progressive relaxation of the TS structure is also consistent with the increasing entropic costs observed along the hydration sequence, since broader and more delocalized proton-transfer pathways require a greater structural arrangement.

**Fig. 4:**
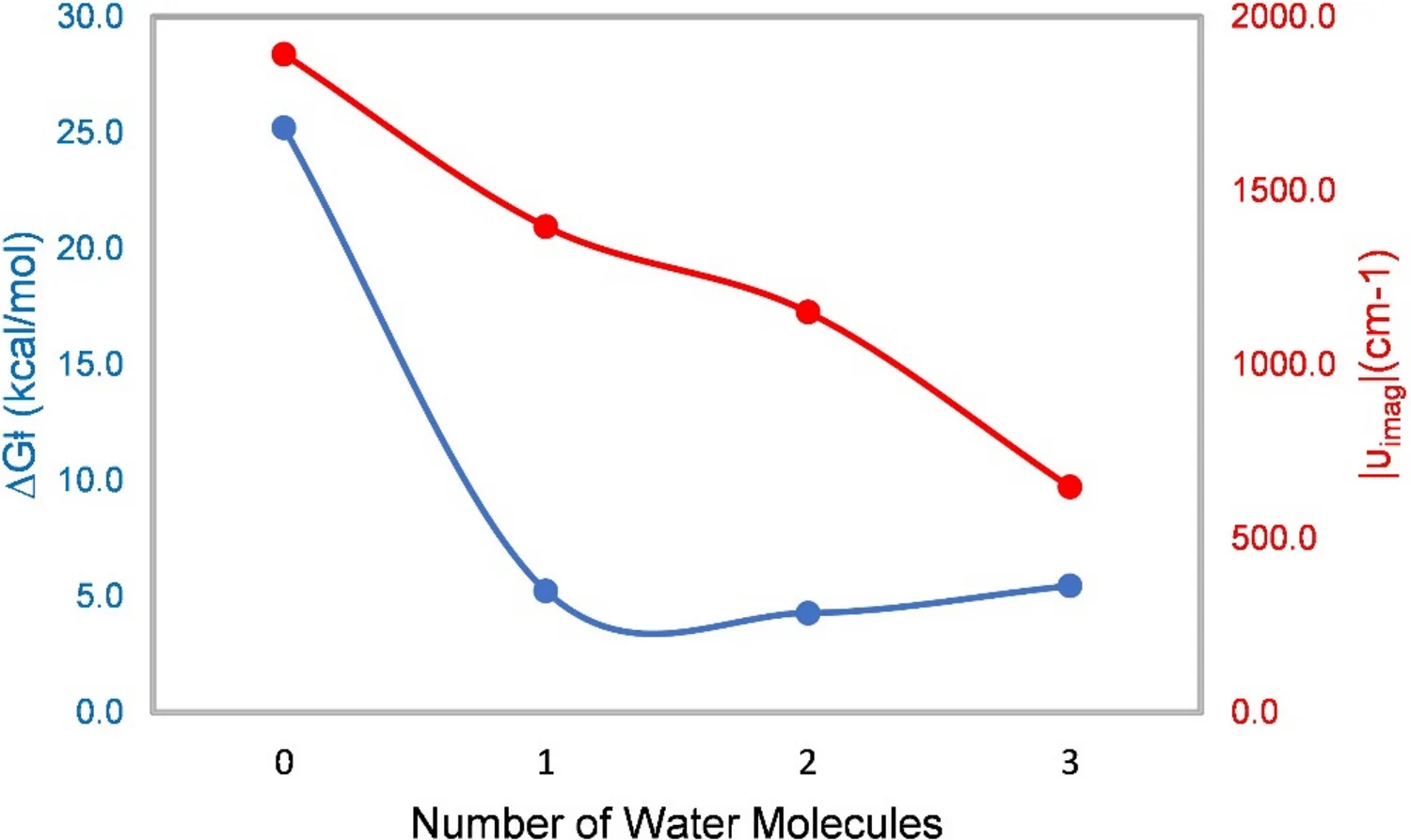
Relationship between the activation free energy (∆*G*^‡^) and the magnitude of the imaginary frequency (|*ν*_imag_|) as a function of the number of water molecules in the hydrated thymine complexes calculated at the M06-2X/def2-TZVP level

The relationship between the activation barriers and the magnitude of the imaginary frequencies along the hydration sequence is summarized in Fig. 4. The progressive decrease in |*ν*_imag_| indicates a gradual relaxation of the transition-state structure upon hydration. However, although the trihydrated system exhibits the lowest |*ν*_imag_| value, its activation barrier slightly increases relative to the bihydrated complex, suggesting that transition-state relaxation alone does not fully determine the proton-transfer energetics.

The non-systematic behavior is observed with the addition of the third water molecule, where the barrier increases to 5.5 kcal/mol. While the bihydrated cluster appears to provide a particularly favorable arrangement for proton transfer among the investigated clusters, the third molecule likely introduces a competitive stabilization of the enol-reactant complex. This stabilization, previously identified through QTAIM and electronic analysis as a result of enhanced cooperativity [15], increases the energy required to reorganize the H-bonded network into the TS configuration. Therefore, although the additional water molecule enhances the overall stabilization, it also increases the structural reorganization required to reach the TS geometry. This interpretation is supported by the thermodynamic decomposition of the activation barriers, where the trihydrated system exhibits the largest entropic contribution among the hydrated complexes. As a consequence, both the *k*_TST_ and tunneling-corrected rate constants decrease relative to the bihydrated system. In particular, the *k*_TST_ values decrease by approximately one order of magnitude, while the corrected rate constants decrease by slightly more than one order of magnitude.

## Structural and intermolecular features of the hydrated complexes

To provide additional insight into the structural and intermolecular factors associated with the proton-transfer process, we integrated the HOMA with the QTAIM. This synergy reveals how the microhydration environment modulates the balance between cyclic π-conjugation patterns and intermolecular H-bonding. HOMA, electron density parameters, and interaction energies for the thymine complexes are summarized in Table 2.

**Table 2:** HOMA, average electron density in H-bond (in a.u.), and average estimated interaction energy, ∆E*_int_ (in kcal/mol) for the thymine-water complexes

System	Form	HOMA	avg ρH-bond	avg ∆E*_int_
Bare thymine	Enol	0.49	–	
	Keto	0.64	–	
Monohydrated	Enol	0.41	0.060	− 14.0
	Keto	0.49	0.025	− 5.7
Bihydrated	Enol	0.65	0.049	− 11.3
	Keto	0.56	0.036	− 8.3
Trihydrated	Enol	0.47	0.048	− 11.2
	Keto	0.52	0.036	− 8.4

avg ∆E*_int_ estimated with ∆E*_int_ = − 236.25 avg ρH-bond + 0.1434 [30]

We now consider the transition from the bihydrated to the trihydrated system. In the bihydrated system, the enol tautomer reaches its maximum aromaticity (HOMA = 0.65), which, together with strong H-bonds, i.e., ∆E*_int_ = −11.3 kcal/mol, creates a favorable environment for the proton transfer with a low activation free energy barrier (Δ*G*‡ = 4.3 kcal/mol at the M06-2X level). Interestingly, the monohydrated system presents the strongest estimated average H-bond interaction energy among the hydrated complexes (avg ∆E*_int_ = − 14.0 kcal/mol), but does not exhibit the lowest activation barrier. This result suggests that the proton-transfer efficiency is not determined exclusively by H-bond strength, but also by the geometric arrangement of the hydration shell and its ability to support an efficient proton-transfer pathway.

However, in the trihydrated system, a different effect is observed. The aromaticity of the enol form drops significantly to 0.47, the lowest value among all studied systems. Although this result suggests a reduction in the degree of cyclic π-conjugation inferred from the molecular geometry, the system remains highly stabilized through intense intermolecular interactions, with a total interaction energy that outweighs the aromatic gain. The QTAIM analysis confirms that the third water molecule strengthens the overall H-bond stabilization, particularly in the keto form, where avg ρH-bond increases from 0.024 to 0.036.

These results indicate that the increase in stabilization does not necessarily correlate with a lower activation barrier. Instead, the balance between intermolecular stabilization, geometric π-conjugation effects, and structural flexibility appears to govern the proton-transfer energetics.

This electronic reorganization may contribute to the increase in the activation energy to 5.5 kcal/mol. The additional water molecule promotes stronger intermolecular stabilization at the expense of part of the π-conjugation of the pyrimidine ring. As a consequence, the enol-reactant complex becomes structurally more stabilized and requires greater reorganization to reach the transition-state geometry, leading to a reduction in the proton-transfer rate relative to the bihydrated system.

In this sense, the bihydrated complex appears to provide a favorable balance between intermolecular stabilization, cyclic conjugation effects, and geometric organization, resulting in a highly efficient proton-transfer pathway among the investigated hydrated systems.

## Conclusions

In conclusion, M06-2X free energy calculations indicate that the two-water cluster exhibits the lowest activation barrier among the investigated systems. Benchmark electronic calculations at higher levels of theory suggest that energetic differences among hydrated clusters are small and method dependent. The key conclusion is that sequential microhydration dramatically facilitates proton transfer relative to isolated thymine, with mono-, bi-, and trihydrated proton wires all providing highly efficient pathways. Finally, HOMA and QTAIM analyses show that these energetics arise from a subtle interplay of intermolecular stabilization, geometric π‑conjugation, and structural reorganization.

Taken together, decomposition of the barrier heights into ∆*H*^‡^ and −*T*∆*S*^‡^ reveals that hydration lowers the enthalpic component, while the entropic contribution increases with the number of water molecules, partially offsetting the stabilization in more hydrated clusters. Although these gas‑phase models do not replicate biological environments directly, they provide molecular‑level insight into how local hydration modulates tautomerization processes in nucleobases.

## Supplementary Information

Below is the link to the electronic supplementary material.

ESM 1 DOCX (24.0 KB)

## Data Availability

No datasets were generated or analysed during the current study.

## References

[1] Watson JD, Crick FHC (1953) Molecular structure of nucleic acids: a structure for deoxyribose nucleic acid. Nature 171:737–738. 10.1038/171737a0 13054692

[2] Brovarets’ OO, Hovorun DM (2019) Atomistic mechanisms of the double proton transfer in the H-bonded nucleobase pairs: QM/QTAIM computational lessons. J Biomol Struct Dyn 37:1880–1907. 10.1080/07391102.2018.1467795 29676661

[3] Slocombe L, Al-Khalili JS, Sacchi M (2021) Quantum and classical effects in DNA point mutations: Watson-Crick tautomerism in AT and GC base pairs. Phys Chem Chem Phys 23:4141–4150. 10.1039/D0CP05781A 33533770

[4] Hossieni H (2026) Mutation in DNA: a quantum mechanical non-adiabatic model. Chem Phys 603:113074. 10.1016/j.chemphys.2025.113074

[5] Vinje J, Falck M, Mazzola F et al (2017) Tautomerization of thymine using ultraviolet light. Langmuir 33:9666–9672. 10.1021/acs.langmuir.7b02473 28835097

[6] Cerón-Carrasco JP, Requena A, Perpète EA et al (2009) Double proton transfer mechanism in the adenine–uracil base pair and spontaneous mutation in RNA duplex. Chem Phys Lett 484:64–68. 10.1016/j.cplett.2009.11.004

[7] Deepa P, Kolandaivel P (2008) Studies on tautomeric forms of guanine-cytosine base pairs of nucleic acids and their interactions with water molecules. J Biomol Struct Dyn 25:733–746. 10.1080/07391102.2008.10507217 18399705

[8] Rejnek J, Hanus M, Kabeláč M et al (2005) Correlated ab initio study of nucleic acid bases and their tautomers in the gas phase, in a microhydrated environment and in aqueous solution. Part 4. Uracil and thymine. Phys Chem Chem Phys 7:2006–2017. 10.1039/B501499A 19787906

[9] Zelený T, Hobza P, Kabeláč M (2009) Microhydration of guanine⋯cytosine base pairs, a theoretical study on the role of water in stability, structure and tautomeric equilibrium. Phys Chem Chem Phys 11:3430. 10.1039/b819350a 19421545

[10] Danilov VI, van Mourik T, Poltev VI (2006) Modeling of the ‘hydration shell’ of uracil and thymine in small water clusters by DFT and MP2 methods. Chem Phys Lett 429:255–260. 10.1016/j.cplett.2006.08.035

[11] Poltev VI, Malenkov GG, Gonzalez EJ et al (1996) Modeling DNA hydration: comparison of calculated and experimental hydration properties of nuclic acid bases. J Biomol Struct Dyn 13:717–725. 10.1080/07391102.1996.10508884 8906892

[12] Kim H-S, Ahn D-S, Chung S-Y et al (2007) Tautomerization of adenine facilitated by water: computational study of microsolvation. J Phys Chem A 111:8007–8012. 10.1021/jp074229d 17637049

[13] Stoyanova N, Enchev V (2022) Tautomerism of cytosine, cytidine, and deoxycytidine: proton transfer through water bridges. Int J Quantum Chem. 10.1002/qua.26958

[14] Würmel J, Simmie JM (2024) Kinetics of direct and water‐mediated tautomerization reactions of nucleobases at low temperatures ⩽200 K. Int J Chem Kinet 56:105–116. 10.1002/kin.21696

[15] Queiroz MH, Alves TV, Rivelino R (2026) Cooperative and stabilization effects in hydrogen-bonded chains of microhydrated thymine: a QTAIM and TD-DFT study. Chem Phys 602:112976. 10.1016/j.chemphys.2025.112976

[16] Kunkel TA (2004) DNA replication fidelity. J Biol Chem 279:16895–16898. 10.1074/jbc.R400006200 14988392

[17] Gu J, Leszczynski J (1999) A DFT study of the water-assisted intramolecular proton transfer in the tautomers of adenine. J Phys Chem A 103:2744–2750. 10.1021/jp982713y

[18] Kruszewski J, Krygowski TM (1972) Definition of aromaticity basing on the harmonic oscillator model. Tetrahedron Lett 13:3839–3842. 10.1016/S0040-4039(01)94175-9

[19] Krygowski TM, Szatylowicz H, Stasyuk OA et al (2014) Aromaticity from the viewpoint of molecular geometry: application to planar systems. Chem Rev 114:6383–6422. 10.1021/cr400252h 24779633

[20] Bader RFW (1985) Atoms in molecules. Acc Chem Res 18:9–15. 10.1021/ar00109a003

[21] Bader RFW (2009) Bond paths are not chemical bonds. J Phys Chem A 113:10391–10396. 10.1021/jp906341r 19722600

[22] Bader RFW (1991) A quantum theory of molecular structure and its applications. Chem Rev 91:893–928. 10.1021/cr00005a013

[23] Frisch MJ; TGW; SHB; SGE; RMA; CJR; SG; BV; MB; PGA; et al. (2009) Gaussian 09, Revision D.01

[24] Zhao Y, Truhlar DG (2008) The M06 suite of density functionals for main group thermochemistry, thermochemical kinetics, noncovalent interactions, excited states, and transition elements: two new functionals and systematic testing of four M06-class functionals and 12 other functionals. Theor Chem Acc 120:215–241. 10.1007/s00214-007-0310-x

[25] Weigend F, Ahlrichs R (2005) Balanced basis sets of split valence, triple zeta valence and quadruple zeta valence quality for H to Rn: design and assessment of accuracy. Phys Chem Chem Phys 7:3297. 10.1039/b508541a 16240044

[26] Contreras-García J, Johnson ER, Keinan S et al (2011) NCIPLOT: a program for plotting noncovalent interaction regions. J Chem Theory Comput 7:625–632. 10.1021/ct100641a 21516178 PMC3080048

[27] Eyring H (1935) The activated complex in chemical reactions. J Chem Phys 3:107–115. 10.1063/1.1749604

[28] Truhlar DG, Garrett BC, Klippenstein SJ (1996) Current status of transition-state theory. J Phys Chem 100:12771–12800. 10.1021/jp953748q

[29] Dobrowolski JCz (2019) Three queries about the HOMA index. ACS Omega 4:18699–18710. 10.1021/acsomega.9b02628 31737831 PMC6854575

[30] Queiroz MH, Santos SA, Nascimento JL et al (2025) Evaluating interaction energy versus electron density relationships to estimate inter and intramolecular H-bonding. J Mol Graph Model 134:108895. 10.1016/j.jmgm.2024.108895 39454274

