# Supplementary material for: Effects of sequential microhydration on thymine tautomerization
Source: J Mol Model. 2026 Aug 29;32(9):335. doi: 10.1007/s00894-026-06916-z (PMC13526113; doi:10.1007/s00894-026-06916-z)
Supplement: Supplementary file 1 [file 894_2026_6916_MOESM1_ESM.docx]

Supporting Information

**Effects of Sequential Microhydration on Thymine Tautomerization**

Murillo H. Queiroz^1^*, Tiago V. Alves^1^, Roberto Rivelino^2^

^1^ Departamento de Físico-Química, Instituto de Química, Universidade Federal da Bahia Rua Barão de Jeremoabo, 147, 40170-115, Salvador, Bahia, Brazil.

^2^Instituto de Física, Universidade Federal da Bahia, 40210-340 Salvador, Bahia, Brazil.

**Table S1.** Electronic activation barriers (in kcal/mol) calculated at the M06-2X/def2-TZVP, CCSD(T)//M06-2X/def2-TZVP, and MP2//M06-2X/def2-TZVP levels for the thymine tautomerization process under different hydration conditions.

| System | M06-2X/def2-TZVP | CCSD(T)//M06-2X/def2-TZVP | MP2//M06-2X/def2-TZVP |
| --- | --- | --- | --- |
| Bare | 27.5 | 27 | 25.3 |
| Monohydrated | 7.8 | 9.1 | 7.5 |
| Bihydrated | 7.3 | 10.4 | 8.5 |
| Trihydrated | 7.8 | 11.1 | 8.9 |

**Table S2.** Cartesian geometries of all complexes optimized at M06-2X/def2-TZVP.

| bare thymine -keto | bare thymine - TS enol→keto |
| --- | --- |
| 6 2.501459000 2.049213000 -0.042704000  6 1.748394000 0.761024000 0.017446000  6 2.318819000 -0.450801000 0.049383000  7 1.590694000 -1.616343000 0.107224000  6 0.212970000 -1.656521000 0.139075000  7 -0.360724000 -0.403960000 0.106725000  1 -1.371351000 -0.384034000 0.128707000  6 0.284663000 0.832481000 0.046935000  8 -0.354376000 1.857699000 0.022622000  8 -0.418007000 -2.683230000 0.190147000  1 2.045332000 -2.514277000 0.128681000  1 3.393056000 -0.581234000 0.031844000  1 3.575925000 1.872322000 -0.057670000  1 2.221699000 2.614236000 -0.932693000  1 2.255909000 2.673964000 0.816999000 | 6 2.494955000 2.002664000 -0.073466000  6 1.707473000 0.733611000 -0.005885000  6 2.222300000 -0.492406000 -0.259460000  7 1.461960000 -1.621840000 -0.184295000  6 0.179200000 -1.540655000 0.146860000  7 -0.410241000 -0.370986000 0.413779000  1 -1.388540000 -0.332809000 0.666482000  6 0.299620000 0.856389000 0.359850000  8 -0.280856000 1.884899000 0.610446000  8 -0.307685000 -2.714756000 0.150828000  1 0.977359000 -2.843609000 -0.185032000  1 3.261349000 -0.626579000 -0.533214000  1 2.474213000 2.519479000 0.887097000  1 3.530392000 1.800420000 -0.343805000  1 2.064145000 2.685407000 -0.807349000 |
| monohydrated - keto | monohydrated - enol |
| 6 -3.178423000 0.412337000 -0.015435000  6 -1.768882000 -0.079327000 -0.001802000  6 -1.416899000 -1.370730000 -0.008228000  7 -0.101945000 -1.778056000 0.005470000  6 0.959385000 -0.907565000 0.021811000  7 0.598044000 0.412205000 0.025575000  1 1.372595000 1.078870000 0.030363000  6 -0.700521000 0.926302000 0.019897000  8 -0.891055000 2.118838000 0.032429000  8 2.112440000 -1.300698000 0.032276000  1 0.143494000 -2.754509000 -0.000982000  1 -2.146380000 -2.169988000 -0.024759000  1 -3.881570000 -0.418983000 -0.039752000  1 -3.378274000 1.022049000 0.866694000  1 -3.350529000 1.050207000 -0.883328000  8 3.339093000 1.181742000 -0.079351000  1 4.039315000 1.484838000 0.501812000  1 3.310296000 0.213241000 -0.018025000 | 6 -3.396190000 0.740901000 0.002234000  6 -1.999447000 0.217645000 0.013217000  6 -1.688661000 -1.083880000 0.005236000  7 -0.370688000 -1.492439000 0.016635000  6 0.615333000 -0.566709000 0.029558000  7 0.407399000 0.707766000 0.037045000  1 2.202560000 1.549579000 -0.031533000  6 -0.897391000 1.203326000 0.035312000  8 -1.113794000 2.395247000 0.051716000  8 1.827311000 -1.090353000 0.034318000  1 -0.112601000 -2.465367000 0.006251000  1 -2.425090000 -1.875896000 -0.010653000  1 -4.124131000 -0.069087000 -0.029278000  1 -3.575723000 1.352403000 0.887638000  1 -3.544798000 1.393490000 -0.859077000  8 3.118975000 1.212041000 -0.071991000  1 3.631426000 1.679723000 0.591547000  1 2.489730000 -0.349173000 0.032352000 |
| monohydrated - TS enol→keto |  |
| 6 -3.293589000 0.692622000 0.008406000  6 -1.894784000 0.173541000 0.008283000  6 -1.577439000 -1.126005000 0.011421000  7 -0.260804000 -1.543103000 0.012163000  6 0.753629000 -0.645279000 0.001751000  7 0.494248000 0.655380000 -0.003748000  1 1.735005000 1.242334000 -0.035607000  6 -0.798763000 1.164034000 0.006939000  8 -1.006497000 2.357320000 0.016572000  8 1.951301000 -1.085381000 -0.002027000  1 -0.011254000 -2.518547000 0.009768000  1 -2.315998000 -1.916468000 0.014158000  1 -4.017467000 -0.121412000 0.013372000  1 -3.460059000 1.327297000 0.879818000  1 -3.463380000 1.318981000 -0.868425000  8 2.872968000 1.101831000 -0.095019000  1 3.324634000 1.463090000 0.673114000  1 2.652057000 -0.061489000 -0.024318000 |  |
| bare thymine- enol | bihydrated - keto |
| 6 3.509351000 4.490904000 -0.861971000  6 2.870836000 3.194692000 -0.484584000  6 3.548927000 2.081833000 -0.115857000  7 2.994978000 0.878586000 0.235458000  6 1.715760000 0.820073000 0.207438000  7 0.923379000 1.866627000 -0.144475000  1 -0.085319000 1.797054000 -0.160960000  6 1.424465000 3.127940000 -0.517441000  8 0.654372000 4.012858000 -0.820546000  1 4.633098000 2.102628000 -0.082793000  1 4.594129000 4.420149000 -0.797304000  1 3.231181000 4.774279000 -1.878347000  1 3.165345000 5.293112000 -0.207203000  8 1.119669000 -0.331388000 0.542192000  1 0.160749000 -0.270978000 0.489692000 | 6 -2.577231000 1.885118000 -0.039327000  6 -1.334619000 1.059710000 0.030896000  6 -0.091306000 1.559506000 0.100658000  7 1.036541000 0.778485000 0.164869000  6 0.988174000 -0.582414000 0.155284000  7 -0.279495000 -1.100272000 0.086956000  1 -0.346416000 -2.109050000 0.080299000  6 -1.484822000 -0.393690000 0.023589000  8 -2.537110000 -0.986127000 -0.033008000  8 1.975808000 -1.301240000 0.202093000  1 1.961546000 1.221401000 0.230406000  1 0.100383000 2.624924000 0.108469000  1 -2.339364000 2.947853000 -0.031655000  1 -3.139276000 1.653226000 -0.944935000  1 -3.232191000 1.661052000 0.803711000  8 3.579138000 1.990438000 0.250507000  1 3.963902000 2.398284000 1.029625000  1 4.124319000 1.198639000 0.055537000  8 4.583220000 -0.483888000 -0.111621000  1 4.971884000 -0.892951000 -0.887543000  1 3.708764000 -0.900100000 0.021086000 |
| bihydrated - TS enol→keto | bihydrated - enol |
| 6 -2.691750000 1.528691000 -0.082676000  6 -1.425720000 0.739340000 -0.012565000  6 -0.188072000 1.274652000 0.016850000  7 0.984620000 0.568227000 0.084488000  6 0.927173000 -0.760832000 0.113748000  7 -0.295849000 -1.359505000 0.088220000  1 -0.312642000 -2.370113000 0.113448000  6 -1.533295000 -0.709438000 0.029128000  8 -2.560733000 -1.351123000 0.014630000  8 1.938990000 -1.519072000 0.163940000  1 2.168552000 1.237004000 0.168595000  1 -0.058631000 2.351674000 -0.013812000  1 -2.482842000 2.597573000 -0.100357000  1 -3.261525000 1.263967000 -0.974718000  1 -3.329497000 1.305407000 0.774029000  8 3.215057000 1.680533000 0.180214000  1 3.394159000 2.140549000 1.004702000  1 3.832493000 0.604070000 0.072408000  8 4.123683000 -0.514629000 -0.011009000  1 4.564328000 -0.728080000 -0.837998000  1 3.111454000 -1.021392000 0.060482000 | 6 -2.468804000 1.645779000 -0.096312000  6 -1.228106000 0.817189000 -0.031692000  6 0.028046000 1.314915000 -0.021483000  7 1.186595000 0.578300000 0.039216000  6 1.048063000 -0.714967000 0.078353000  7 -0.166258000 -1.311021000 0.073953000  1 -0.207423000 -2.321044000 0.105887000  6 -1.387192000 -0.625344000 0.024156000  8 -2.431826000 -1.237717000 0.027285000  8 2.050058000 -1.554494000 0.125924000  1 2.671456000 1.583969000 0.199433000  1 0.181651000 2.388506000 -0.064714000  1 -2.226420000 2.707216000 -0.123660000  1 -3.053799000 1.391464000 -0.981569000  1 -3.105617000 1.447274000 0.767170000  8 3.559438000 2.014356000 0.235350000  1 3.618459000 2.480607000 1.072398000  1 4.316376000 0.489996000 0.041231000  8 4.395089000 -0.485499000 -0.070234000  1 4.884192000 -0.647035000 -0.880622000  1 2.948342000 -1.098840000 0.054329000 |
| trihydrated -keto | trihydrated - TS enol→keto |
| 6 2.362080000 2.155404000 0.342163000  6 1.628033000 0.874877000 0.118380000  6 2.188148000 -0.340649000 0.170680000  7 1.466929000 -1.493970000 -0.038452000  6 0.122688000 -1.501398000 -0.318767000  7 -0.441393000 -0.266453000 -0.394512000  1 -1.445863000 -0.193221000 -0.647484000  6 0.194886000 0.952512000 -0.179073000  8 -0.428890000 1.990644000 -0.242327000  8 -0.488103000 -2.546533000 -0.480174000  1 1.902123000 -2.400100000 0.017226000  1 3.239215000 -0.486512000 0.383035000  1 3.411187000 1.970178000 0.568597000  1 2.295997000 2.792239000 -0.540969000  1 1.911678000 2.711299000 1.165363000  8 -4.965253000 -0.939529000 0.064818000  1 -4.381695000 -1.721863000 0.134565000  1 -5.694601000 -1.201117000 -0.501786000  8 -2.957408000 0.527540000 -1.069538000  1 -2.784945000 1.416084000 -0.739803000  1 -3.770562000 0.184762000 -0.649740000  8 -3.164101000 -3.012193000 0.136465000  1 -3.040456000 -3.559518000 0.914998000  1 -2.267976000 -2.836701000 -0.205862000 | 6 2.215066000 2.110216000 0.314876000  6 1.500471000 0.815325000 0.115684000  6 2.097767000 -0.383854000 0.101153000  7 1.373119000 -1.535960000 -0.083283000  6 0.014060000 -1.514350000 -0.268925000  7 -0.612208000 -0.348462000 -0.294976000  1 -2.031170000 0.094623000 -0.806710000  6 0.045142000 0.845804000 -0.087418000  8 -0.578430000 1.902425000 -0.084922000  8 -0.558502000 -2.630309000 -0.400354000  1 1.809085000 -2.442812000 -0.068800000  1 3.163881000 -0.515011000 0.234736000  1 3.286674000 1.955310000 0.438760000  1 2.044455000 2.771592000 -0.535674000  1 1.824986000 2.626966000 1.192932000  8 -4.465864000 -1.032620000 -0.157790000  1 -3.739158000 -1.924433000 -0.001157000  1 -5.143727000 -1.249872000 -0.804305000  8 -2.915466000 0.584892000 -1.015155000  1 -2.731810000 1.461013000 -0.646323000  1 -3.832739000 -0.187238000 -0.528272000  8 -2.923632000 -2.847789000 0.144261000  1 -2.940303000 -3.228499000 1.026070000  1 -1.884021000 -2.701474000 -0.142332000 |
| trihydrated - enol |  |
| 6 2.308315000 2.105417000 0.203066000  6 1.592774000 0.800457000 0.101749000  6 2.205116000 -0.387991000 0.027534000  7 1.473508000 -1.551095000 -0.065132000  6 0.118156000 -1.503583000 -0.091027000  7 -0.548263000 -0.394777000 -0.034285000  1 -2.264032000 0.207711000 -0.519867000  6 0.119491000 0.820039000 0.074314000  8 -0.501227000 1.866891000 0.135910000  8 -0.442638000 -2.680502000 -0.179258000  1 1.913699000 -2.455402000 -0.108561000  1 3.279953000 -0.508259000 0.036686000  1 3.388633000 1.965491000 0.217421000  1 2.039912000 2.746706000 -0.637499000  1 2.001630000 2.633646000 1.106721000  8 -4.867240000 -1.167650000 -0.577931000  1 -3.673429000 -2.246423000 -0.152736000  1 -5.326596000 -1.258531000 -1.415543000  8 -3.058585000 0.736871000 -0.729611000  1 -2.807734000 1.614463000 -0.421829000  1 -4.361640000 -0.321838000 -0.618968000  8 -2.953905000 -2.819162000 0.218142000  1 -3.163719000 -2.937794000 1.147894000  1 -1.454501000 -2.639202000 -0.075113000 |  |
